# Thrombin and Factor Xa Hydrolysis of Chromogenic Substrates in the Presence of Sulfated Derivatives of Galactomannan and Galactoglucomannan Natural Gels

**DOI:** 10.3390/pharmaceutics14122678

**Published:** 2022-12-01

**Authors:** Natalia N. Drozd, Svetlana A. Kuznetsova, Yuriy N. Malyar, Aleksandr S. Kazachenko, Valentina S. Borovkova, Yarosvala D. Berezhnaya

**Affiliations:** 1 National Medical Research Center of Hematology, Ministry of Health of the Russian Federation, Novy Zykovskiy Proezd 4, Moscow 125167, Russia; 2 Institute of Chemistry and Chemical Technology, Krasnoyarsk Science Center, Siberian Branch, Russian Academy of Sciences, Akademgorodok 50/24, Krasnoyarsk 660036, Russia; 3 School of Non-Ferrous Metals and Materials Science, Siberian Federal University, pr. Svobodny 79, Krasnoyarsk 660041, Russia; 4 Department of Biological Chemistry with Courses in Medical, Pharmaceutical and Toxicological Chemistry, Krasnoyarsk State Medical University, Ministry of Healthcare of the Russian Federation, ul. Partizana Zheleznyaka 1, Krasnoyarsk 660022, Russia

**Keywords:** galactomannan sulfate, galactoglucomannan sulfate, chromogenic substrate, thrombin, factor Xa, platelet aggregation

## Abstract

Polysaccharides are important structural components of all plant species. Gel-like polysaccharides have found wide application in various fields, including medicine, construction, and the food industry. In the present work, galactomannan and galactoglucomannan gel-like polysaccharides were modified with sulfate groups and their anticoagulant activity was studied. Sulfation with chlorosulfonic acid in pyridine and with sulfamic acid in pyridine and a sulfamic acid–urea deep eutectic solvent were used as synthesis routes. The resulting gel-like polysaccharide sulfates were studied by elemental analysis, Fourier-transform infrared spectroscopy, and gel permeation chromatography. It was established that the anticoagulant effect of sulfated galactoglucomannan (SGGM) and galactomannan (SGM-1 and SGM-2) is related to an independent antithrombin-independent decrease in the amidolytic activity of thrombin and factor Xa. It is shown that the inhibitory activity of SGGM and SGM-2 against the collagen-induced platelet aggregation can be an additional factor in selecting compounds that are most promising for modifying polymer surfaces to ensure resistance to blood clotting.

## 1. Introduction

Polysaccharide (PS) biopolymers are promising raw materials for implantable medical devices due to their hydrophilicity, biodegradability, biocompatibility, nontoxicity to human cells, and low cost [1,2,3]. Owing to reactive functional groups contained in the PS and PS derivative structures, the latter can be used to synthesize films, hydrogels, nanocomposites, and 3D frameworks with desired characteristics. The composition of the materials for cardiovascular devices (vascular stents, vascular grafts, ventricular valves, blood monitors, intravascular endoscopes, catheters for nonsurgical treatment of coronary heart disease, heart–lung machines, extracorporeal membrane oxygenators, hemodialysis equipment, temporary pacemaker electrodes, subcutaneous circulatory support systems, blood circulation lines, intravascular catheters, etc.) resistant to the occurrence of fibrin blood clots on the surface can also include natural bacterial, fungal, algal, vegetable, or animal PSs and PS derivatives [1,4]. 

The use of PSs (chitosan, alginate, hyaluronic acid, chondroitin sulfate, dextran sulfate, heparin, carrageenan, fucoidan, galactomannan, glucan, pectin, pullulan, levan, and cellulose) to modify the surfaces of medical devices helps improve their hemocompatibility and enhance the thromboresistance [5,6,7,8,9]. Some polysaccharides can serve as sources of functional foods used to prevent or treat cardiovascular diseases [10]. The anticoagulant (AC, inhibition of the fibrin clot formation) and antiplatelet activities of natural PSs and their derivatives make them excellent candidates for surface modification [11,12,13,14,15]. One of the mechanisms of the AC activity is inhibition of the activity of coagulation factors by a compound, either directly or via activating plasma inhibitors of serine proteinases of the blood coagulation system (antithrombin (AT) and heparin cofactor II) [16,17]. AT is a plasma inhibitor of serine proteinases of the blood coagulation system. Preparations of anticoagulant heparin widely used in surface modification (unfractionated heparin (UFH) and low-molecular-weight heparin (LMWH)) activate factor IIa (thrombin-inhibiting AT) and factor Xa; these drugs are characterized by the antithrombin (aIIa) and anti-factor Xa (aXa) activities [18,19]. 

Gracher et al. [20] showed that sulfated mannogalactan from oyster mushroom *Pleurotus ostreatoroseus* inhibited thrombin through AT and heparin cofactor II and the ADP-induced platelet aggregation. The AC activity of the sulfated polysaccharide from the marine green alga *Monostroma nitidum*, which contains rhamnose, xylose, glucose, and glucuronic acid residues, was explained by the intense potentiation of the activity of heparin cofactor II during thrombin inhibition, as well as by the acceleration of the thrombin and factor Xa inhibition via the activation of antithrombin [21]. Sulfated α-glycans from sea urchin tissues inhibited thrombin and factor Xa via AT and independently inhibited platelet aggregation [11]. De Araujo et al. [22] found that the polysaccharide extract from the stem bark of *Caesalpinia (Libidibia) ferrea* containing uronic and glucuronic acids inhibited the platelet aggregation induced by ADP and collagen.

The aim of this study is to synthesize sulfated derivatives of natural gel-like galactomannan (GM) and galactoglucomannan (GGM) polysaccharides and analyze their effect on the activity of thrombin and factor Xa with respect to the chromogenic substrates (the amidolytic activity) and the collagen-induced platelet aggregation.

## 2. Materials and Methods

### 2.1. Synthesis of Sulfated Gel-like Polysaccharides

#### 2.1.1. Obtaining a Sulfating Complex

The pyridine and sulfur trioxide complex used in GM sulfation was obtained by the interaction of pyridine with chlorosulfonic acid. To do that, 50 mL of dioxane was placed in a three-necked flask equipped with a thermometer, a mechanical stirrer, and a dropping funnel and subjected to vigorous stirring at a temperature of 5 °C, during which 5 mL of chlorosulfonic acid was added dropwise.

#### 2.1.2. GM Sulfation with Sulfur Trioxide in Pyridine

In the experiments, Sigma-Aldrich guar gum was used. The sulfur trioxide and pyridine complex was added with 1.0 g of guar gum under stirring at a temperature of 80–90 °C for 60–180 min. At the end of the sulfation process, the reaction mass was neutralized with an aqueous solution of sodium (or ammonium) hydroxide to pH 8–9.

#### 2.1.3. Gel-like GM Sulfation in Deep Eutectic Solvent (DES)—Sulfamic Acid: Urea Mixture

The sulfation of GM in DES was provided as described in [23].

#### 2.1.4. GGM Sulfation with Sulfamic Acid in Pyridine

Sawdust (a fraction of 2.0–5.0 mm) of Siberian larch (*Larix Sibirica*) growing in the Krasnoyarsk Territory (Russia) was used as a feedstock. The contents of the main larch wood components (wt %) were 42.2 of cellulose, 28.1 of lignin, 26.7 of hemicelluloses, 2.0 of resins, and 1.0 of ash.

The method described in [24] was used to isolate GM.

For GGM sulfation, 50 mL of pyridine, 6.2 g of sulfamic acid, and 3.8 of urea were placed in a three-necked flask equipped with a thermometer and a mechanical stirrer. The resulting mixture was heated to 50 °C under vigorous stirring and added with 1 g of air-dry GGM. Then, the temperature of the reaction mixture was increased to a fixed value (see the sulfation conditions in Table 1) and stirred at this temperature for 1–3 h. At the end of sulfation, the solvent was decanted, the obtained residue was dissolved in 25 mL of water, and the excess of sulfamic acid was neutralized with 25% aqueous ammonia until neutral.

#### 2.1.5. Dialysis of Sulfated Polysaccharides

To remove low-molecular-weight reaction products and impurities, the obtained product was dialyzed against water in an MFPI MF-503-46 plastic bag (U.S.) with a pore size of 3.5 kDa for 10 h. The dialysis process was intensified using deionized water, which was changed every hour. During dialysis, high-molecular-weight reaction products and impurities consisting of inorganic compounds and low-molecular-weight PS modification products were separated. The purified high-molecular-weight part was then dried in Petri dishes at room temperature.

### 2.2. Methods of the Physicochemical Analysis

#### 2.2.1. Elemental Analysis

The elemental analysis of sulfated guar gum was carried out in a ThermoQuest FlashEA-1112 elemental analyzer (Italy).

#### 2.2.2. Fourier-Transform Infrared Spectroscopy

Fourier-transform infrared (FTIR) spectra of initial and sulfated GG and GGM were recorded in the range of 4000–400 cm^−1^ on a Shimadzu IRTracer-100 FTIR spectrometer (Kyoto, Japan). During the FTIR analysis, a tableted polymer was mixed with KBr (2 mg of the sample per 1000 mg of KBr). The spectral data were processed using the OPUS software (version 5.0).

#### 2.2.3. Gel Permeation Chromatography

The weight-average molecular weight *M*_w_, number-average molecular weight *M*_n_, and polydispersity of the sulfated GGM (SGGM) samples were determined by gel permeation chromatography (GPC) using an Agilent 1260 Infinity II Multi-Detector GPC/SEC system with two detectors: a refractometer (RI) and a viscometer (VS). The separation was made on two Agilent PL aquagel-OH columns. The samples were eluted with an eluent composed of 0.1 M NaNO_3_ and 0.25 g/L of NaN_3_ as a stabilizer (pH 7) at a flow rate 1 mL/min and a sample volume of 100 μL. Polyethylene glycol standards (Agilent, Santa Clara, CA, USA) were used to calibrate the columns. All the samples were dissolved overnight in the mobile phase (1–5 mg/mL) and then filtered through a 0.22-μm Agilent PES membrane filter to remove impurities. The data were collected and processed using the Agilent GPC/SEC MDS software.

### 2.3. Anticoagulant Activity

In this study, blood stabilized with a solution of 0.106 M of sodium citrate from the donors’ cubital vein taken up to the mark in a Sarstedt S -Monovette 5 mL 9NC plastic syringe (Germany) was used; all donors gave written informed consent for the blood and plasma collection and use at the National Research Center for Hematology, Ministry of Health of the Russian Federation. Human platelet-rich plasma (PRP) was obtained by centrifuging blood for 7 min at 150× *g* at room temperature. Platelet-poor plasma (PPP) was obtained by centrifuging blood for 20 min at 1300× *g*. The GM/GGM sulfate solutions for the analysis were prepared using 0.05 M of a Tris-HCl buffer with 0.175 M NaCl (pH 7.4).

#### 2.3.1. The AXa Anticoagulant Activity

The effect of SGM/SGGM on the plasma clotting time was examined routinely by the coagulological method using the ReaClot-heparin kit of reagents (NPO “Renam”, Moscow, Russia) to determine the aXa activity of an anticoagulant (for example, heparin). The method used is based on the ability of small amounts of heparin in the investigated plasma to neutralize the exogenous factor Xa in the presence of antithrombin [25]. The reaction formulation is as follows: PRP (0.058 mL) containing 1.463–146.3 µg/mL of GM/GGM was incubated for 1 min at 37 °C. After adding 0.028 mL of the mixture of factor Xa with phospholipids, the samples were incubated for 3 min at 37 °C. Next, 0.021 mL of the 0.035 M CaCl_2_ solution was added and the time(s) for the occurrence of a fibrin clot was determined with a Minilab-701-M APG2-01 programmable semi-automatic coagulometer (NPO “Emko”, Russia). Using the dependence of the examined effect on the GM/GGM concentration, the 2Reaclot concentrations at which the PRP clotting time was twice as long as for the control without adding the samples were determined. The anti-factor Xa activity of the samples was calculated using plasma from the ReaChrom-Xa kit (NPO “Renam”, Russia) containing LMWH with activity levels of 0.51 and 0.97 U/mL.

#### 2.3.2. Thrombin Hydrolysis of the Chromogenic Substrate

The effect of SGM/SGGM on the thrombin hydrolysis of the chromogenic substrate was estimated routinely using the Renaparin kit of reagents (NPO “Renam”, Russia) by the method described in [26]. To do that, 0.02 mL of a buffer or AT and 0.1 mL of the thrombin solution were added to 0.110 mL of a Tris-HCl buffer (pH 7.4) containing the GM/GGM sulfates (1.8–1800 µg/mL). After incubation for 1 min at 37 °C, 0.1 mL of the chromogenic substrate solution per thrombin was added to the mixture and the optical density variation per minute (A405/min) was recorded on a Bio-Rad SmartSpec Plus spectrophotometer (US). Using the GM/GGM concentration dependences of A405/min, the concentrations of the samples at which the optical density variation was halved ([A405/min:2] mg/mL) compared with the control were determined. As a control with the aIIa activity, UFH (5000 IU/mL; Belmedpreparaty, Minsk, Belarus) was used.

#### 2.3.3. Factor Xa Hydrolysis of the Chromogenic Substrate

The effect of SGM/SGGM on the factor Xa hydrolysis of the chromogenic substrate was established routinely using the ReaChrom-Xa kit of reagents (NPO “Renam”, Russia) by the method from [27]. For this purpose, 0.05 mL of a Tris-HCl buffer containing AT or a buffer and 0.05 mL of the GM/GGM solution (169.44–1694.4 µg/mL) were added to 0.06 mL of the factor Xa solution. After incubation for 5 min at 37 °C, 0.06 mL of the chromogenic substrate solution was added, the optical density variation per minute (A405/min) was recorded on a Bio-Rad SmartSpec Plus spectrophotometer (U.S.), and [A405/min:2] mg/mL was determined. As a control with the aXa activity, the LMWH working standard from the Renaparin kit (aXa = 10 U/mL) was used.

#### 2.3.4. Platelet Aggregation Test

The effect of SGM/SGGM on platelet aggregation was studied using a Chrono-Log Model 500 aggregometer (U.S.) with a recorder detecting changes in the transmission of a light beam in human platelet-rich plasma by the Born’s method [28]. The collagen solution (NPO “Renam”) was used as an aggregation inducer. The platelet-rich human plasma (0.450 mL) was added with 0.045 mL of the GM/GGM solution (the final concentration is 1.67 mg/mL) or a buffer incubated for 1 min at 37 °C and then with 0.045 mL of the collagen solution (NPO “Renam”, the final concentration is 50 µg/mL). The plasma added with 0.045 mL of the 0.05 M Tris-HCl buffer (pH 7.4) containing 0.175 M NaCl was used as a control. The platelet aggregation was determined for 15 min (the transmission of a light beam in human PPP was taken as 100%). The platelet aggregation curve was used to determine the lag phase (min), maximum amplitude (%), slope of the platelet aggregation curve (arb. units/min), and area under it (rel. units ∙ min).

#### 2.3.5. Statistical Processing

The statistical processing of the results was carried out in the Biostat and Statistica programs. To compare the anomalously distributed data, the nonparametric Mann-Whitney U test was used. The results obtained were presented as arithmetic means ± standard errors of the arithmetic means from 3–6 independent determinations. The statistically significant differences between the data series were observed at *p* < 0.05.

## 3. Results

### 3.1. Synthesis and Characterization of the GM/GGM Sulfates

Data on the sulfur content in the obtained GM and GGM sulfates are given in Table 1.

According to the data given in Table 1, sulfation of GM with chlorosulfonic acid in pyridine yields the sulfated product with a sulfur content of up to 13.2 wt %. An increase in both the processing time and temperature causes the growth of the sulfur content and hydrolysis of the polysaccharide primary structure [29,30]. Sulfation of GM with the sulfur trioxide complex in organic solvents leads to partial hydrolysis of the biopolymer macromolecular structure [31,32].

Sulfation of GGM with sulfamic acid in pyridine yields the GGM sulfates with a sulfur content of up to 16.5 wt % at a process temperature of 90 °C and a process time of 2.5 h. The results obtained are comparable with the data on sulfation with sulfamic acid in 1,4-dioxane [24] and DMSO [33].

**Figure 1:**
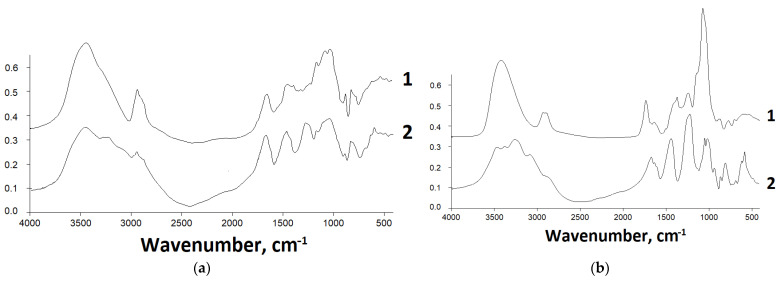
IR spectra for (**a**) GM and (**b**) GGM ((1) initial and (2) sulfated).

The original and sulfated polysaccharides were studied by IR spectroscopy (Figure 1).

According to the IR spectroscopy data (Figure 1), the introduction of a sulfate group into GM and GGM polysaccharide macromolecules leads to the occurrence of absorption bands in the ranges of 1245–1255 and 802–818 cm^−1^, which correspond to the sulfate group vibrations. In addition, a decrease in the intensity of the absorption bands of the hydroxyl group in the range of 3000–3500 cm^−1^ is observed. At the same time, in this region, the peak broadens due to the superposition of the absorption bands of OH- and NH-groups.

**Figure 2:**
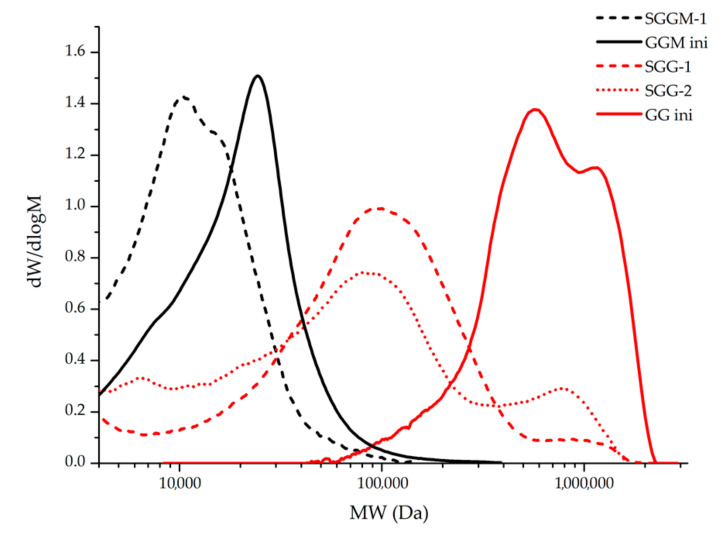
Molecular weight distributions for the initial and sulfated polysaccharides samples.

The initial larch wood GM samples have a low molecular weight, like most plant oligosaccharides. [24]. After sulfation at a temperature of 90 °C for 2.5 h, the molecular weight distribution somewhat changes (Figure 2). In particular, the main peak shifts toward lower molecular weights and the second peak appears, which is most likely indicative of side processes of hydrolysis of the main polysaccharide chain. In addition, the partial depolymerization processes explain a significant increase in the sulfur content (up to 16.5 wt %); in this case, the sulfate groups are added, in particular, at the places of glycosidic bond breaking. The combination of these factors leads to a certain increase in the polydispersity index (up to 1.5).

The initial GM sample has low molecular weight (*M*_w_ ≈ 660 kDa), which is consistent with the data reported in [30,34]. After sulfation for 150 min at 80 °C, the destruction of GM chains and molecular weight redistribution probably occur in the sample: the low-molecular peak of the fraction with MM ≈ 600 kDa disappears and a new peak corresponding to the reaction product with MM ≈130 kDa arises. The high-molecular-weight fraction in the sample also noticeably decreases (see Figure 2). An increase in the sulfation temperature to 80 °C leads to the further degradation of the GM polymer chains with a decrease in the MM of the main fraction of the sulfated product to ~110 kDa, while the high-molecular-weight fraction, on the contrary, somewhat increases, probably due to the higher resistance of molecules to the degradation and an increase in the GM sulfation degree. The molecular weight redistribution leads to an increase in the polydispersity index from 2.75 to 4.78.

### 3.2. Hemocompatibility of the GM/GGM Sulfates

As we showed previously [35], the SGM and SGGM samples did not facilitate the hemolysis of human erythrocytes and, with an increase in their concentration in human blood/plasma, the time of the occurrence of a fibrin clot in the tests of the blood recalcification (BRC) time, activated partial thromboplastin time (APTT), and prothrombin time increased. The AT (aIIa) activity of the direct-acting anticoagulants attained 64.25 ± 7.30 U/mg.

In this work, to establish the mechanism of the AC action of the GGM/GM sulfates (see Table 2), we explored in vitro their effect on the specific chromogenic substrates for thrombin and activated factor Xa (the amidolytic activity of factors) in the reactions with and without AT.

### 3.3. Hemocompatibility of the GM/GGM Sulfates

Most of the blood coagulation factors involved in the conversion of fibrinogen to fibrin are proteolytic enzymes (serine proteases) [36,37]. The activity of such enzymes can also be estimated using the chromogenic substrates. The chromogenic substrate contains a chromophore (p-nitroanilide) attached to a synthetic peptide with an amino acid sequence that mimics the active site interacting with a specific protein of the blood coagulation system. Under the interaction of the enzyme with a chromogenic substrate, p-nitroanilide is released and a yellow color of the solution develops, which can be measured by the absorption at 405 nm. The color intensity is directly proportional to the tested enzyme activity [37,38,39].

The method used in this work is based on the ability of the AT-heparin complex (or any other direct anticoagulant) to neutralize the activity of thrombin (factor IIa) and factor Xa [26,40]. The activity of the anticoagulant sample is determined by adding excess AT and factor Xa or IIa. In this case, factor Xa or IIa is inhibited by either the AT-anticoagulant complex or the anticoagulant. The remaining amount of factor Xa or IIa catalyzes the detachment of para-nitroaniline from the chromogenic substrates.

**Figure 3:**
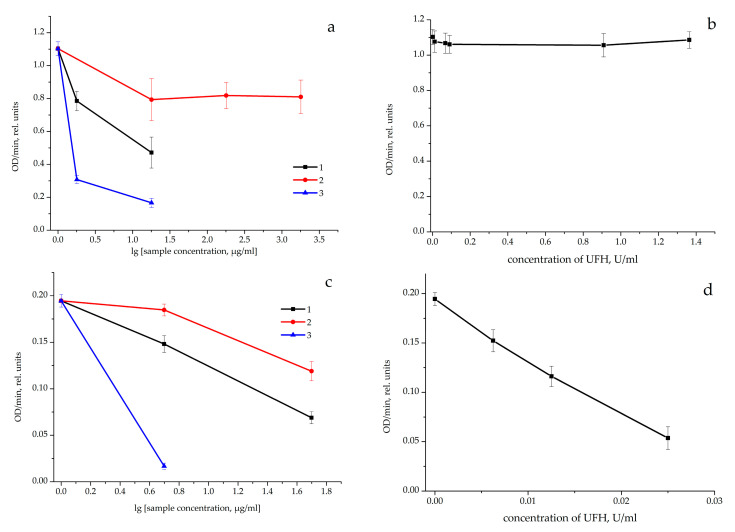
Effect of (**a**,**c**) the GGM/GM sulfates and (**b**,**d**) UFH on the optical density variation (OD/min, rel. units) of the solution containing (**a**,**b**) thrombin and chromogenic substrate (*n* = 3–4) or (**c**,**d**) thrombin, antithrombin, and chromogenic substrate (*n* = 3–6). (1) SGGM, (2) SGM-1, and (3) SGM-2.

The incubation of thrombin with the GGM/GM sulfates at a concentration of 18 µg/mL reduced the rate of hydrolysis of the synthetic chromogenic substrate for thrombin (without adding AT) by factors of 2.34 (A405/min 0.472 ± 0.094 arb. units) and 6.79 (A405/min 0.167 ± 0.028 arb. units), respectively, compared with the control (A405/min in the control 1.103 ± 0.042 rel. units) (Figure 3a). The concentrations of [A405/min:2] SGGM and SGM-2 in the reaction of thrombin with the chromogenic substrate were 19 ± 7.2 and 1.4 ± 0.2 µg/mL, respectively. Adding SGM-1 (with a lower molecular weight and the aIIa activity lower by a factor of 5.55 than in SGM-2) to the incubation mixture did not reduce the reaction rate. The [A405/min:2] concentrations for SGM-1 and UFH could not be detected (Figure 3a,b). As is known, the AC mechanism of the UFH action is related to the AT-mediated inhibition of the thrombin activity [18].

The incubation of thrombin with AT and GGM/GM sulfates at concentrations of 5 µg/mL and 50 µg/mL reduced the rate of hydrolysis of the synthetic chromogenic substrate for thrombin compared with the control (A405/min in the control 0.1945 ± 0.0067 rel. units) (Figure 3c). The SGGM, SGM-1, and SGM-2 concentrations [A405/min:2] attained 28.1 ± 5.5, 70.2 ± 10.7, and 3.1 ± 0.38 µg/mL, respectively. For UFH, the concentration [A405/min:2] mg/mL was 0.0161 ± 0.0016 U/mL (Figure 3d). The incubation of thrombin with AT and the GGM/GM samples also reduced the rate of hydrolysis of the chromogenic substrate.

**Figure 4:**
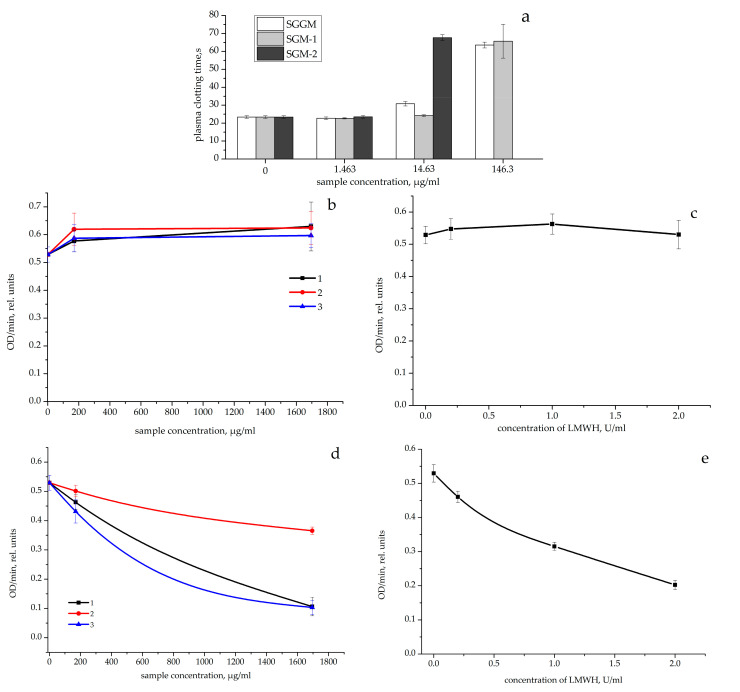
Effect of the GGM/GM sulfates on the plasma clotting time (s) in the ReaClot-Heparin test (**a**) (*n* = 6). Effect of (**b**,**d**) the GGM/GM sulfates and (**c**,**e**) LMWH on the optical density variation (OD/min, rel. units) in the solution containing factor Xa, the chromogenic substrate (**b**,**c**; *n* = 6) or factor Xa, the antithrombin, chromogenic substrate (**d**,**e**; *n* = 6). (1) SGGM, (2) SGM-1, and (3) SGM-2.

Prior to analyzing the effect of the samples on the amidolytic activity of factor Xa, the effect of the GGM/GM sulfates on the plasma coagulation was studied in the Reaclot-heparin test with factor Xa as a reactive. As the sample concentration increased (1.463–146.3 µg/mL), the time for the occurrence of a fibrin clot in plasma significantly increased compared with the control (Figure 4a). The 2Reaclot concentrations for the SGGM, SGM-1, and SGM-2 samples were 59.9 ± 28.5, 89.7 ± 51.8, and 8.9 ± 1.5 µg/mL, respectively; their anti-factor Xa activities attained 8.64 ± 4.11, 5.79 ± 2.28, and 52.50 ± 10.15 U/mg, respectively.

How do SGGM/SGM with the aXa activity affect the amidolytic activity of factor Xa: directly or indirectly via AT? The incubation of factor Xa with SGGM/SGM (169.44 and 1694.4 µg/mL) or LMWH (0.2–2 U/mL) did not change the rate of hydrolysis of the chromogenic substrate for factor Xa without adding AT (Figure 4b,c).

The factor Xa incubation with AT and the SGGM/SGM samples (169.44 and 1694.4 µg/mL) reduced the rate of hydrolysis of the chromogenic substrate for factor Xa compared with the control (A405/min in the control 0.5293 ± 0.0256 rel. units, Figure 4d). The SGGM, SGM-1, and SGM-2 concentrations [A405/min:2] attained 991.7 ± 89.1, 2500 ± 174.1, and 876.7 ± 137.6 µg/mL, respectively. The growth of the LMWH concentration in the incubation mixture with factor Xa and AT led to a decrease in the reaction rate (Figure 4e) and the concentration [A405/min:2] was 1.358 ± 0.1051 U/mL. Thus, the SGGM/SGM samples alone did not affect the amidolytic activity of factor Xa. The concentrations [A405/min:2] of the SGGM and SGM-2 samples in the reaction with AT did not differ, although the aXa activity of SGM-2 was higher by a factor of 6.

The AT-mediated inhibition of the thrombin and factor Xa activity by the polysaccharide sulfates isolated from both plant and animal sources was demonstrated by different authors [6,11,18,20,21].

The effect of the samples on the platelet aggregation should be analyzed, since the antiplatelet activity, like the AC activity, prevents blood clotting [11,12,13,14,15]. Platelets play a key role in stopping bleeding at the damage of a vascular wall. The resulting “platelet plug” is caused by a complex series of reactions and the three main phases of its formation can be distinguished: adhesion, aggregation, and release. As we showed previously, the GM and GGM sulfate samples with a concentration of 2 mg/mL significantly reduced the platelet aggregation induced by ADP, which activates purine receptors on the platelet membrane [41] by factors of 1.43 and 2.19, respectively, compared with the control [35]. In this work, we analyzed the SGGM/SGM effect on the human platelet aggregation induced by collagen, which triggers the platelet aggregation, as well as the synthesis and secretion of thromboxane A2 from platelet granules, and interacts with platelet membrane glycoprotein receptors GPVI and GPIa/IIa [42]. Our interest in collagen is due to the fact that, unlike ADP, this compound is a strong inducer, which causes platelet aggregation. The PRP incubation with SGGM, SGM-1, and SGM-2 (1.67 mg/mL) weakened the collagen-induced platelet aggregation by factors of 2.94, 3.24, and 2.56, respectively. In addition, the significant lag-phase shortening (by factors of 2.65, 5.21, and 7.19) and a decrease in the slope (by factors of 2.67, 4.01, and 4.67) and a decrease in the area (by factors of 2.77, 3.15, and 1.95) were observed in the platelet aggregation curve (Figure 3a–d) compared with the control. The platelet aggregation with collagen has a pronounced latent phase during which phospholipase C is activated. Depending on the concentration of the reagent used, the length of this phase can be 5–7 min. When this period is over, the processes occur in platelets that lead to the formation of secondary intermediaries, which results in the development of the secretion of platelet granules and the synthesis of thromboxane A2 accompanied by the strong intensification of the interthrombocyte interaction) [43]. To clarify why the incubation with the GM and GM sulfates leads to a decrease in the lag phase, it is necessary to thoroughly investigate the antiplatelet effect of the samples during aggregation of the collagen-induced platelet. Jaffe and Deykin described in detail the fibrillogenesis stages during aggregation with collagen. It was assumed that the lag phase consists of at least three separate events: (i) aggregation of tropocollagen with the formation of microfibrils, (ii) platelet adhesion to collagen microfibrils, and (iii) release of the platelet components and platelet aggregation [44]. In later studies [45,46] the authors showed that the lag-phase length in the collagen-induced platelet aggregation depends on the size of aggregates (in fibrillogenesis) in the experiments with sulfonated chitosan or alginate. The authors recommended to use sulfonated chitosan and alginate as analogs of the extracellular matrix for creating biomimetic materials. For this purpose, the authors proposed to use alginate, although it did not inhibit the collagen-induced platelet aggregation. In addition, Ferroni et al. [47] reported a significant decrease in the lag phase with the collagen-induced platelet aggregation in patients receiving the antiplatelet agent aspirin.

De Araujo et al. [22] found the level of the collagen-induced platelet aggregation of the same order (24%) during the incubation of PRP with a polysaccharide extract isolated from the bark of the trunk of the Brazilian iron tree *Caesalpinia (Libidibia) ferrea*. Silver et al. showed that the polysaccharide glycosaminoglycan chondroitin-6-sulfate inhibits collagen fibrillogenesis and collagen-induced platelet aggregation [48]. The authors put forward the idea that the complex of chondroitin-6-sulfate with collagen can be used as a blood-compatible material.

**Figure 5:**
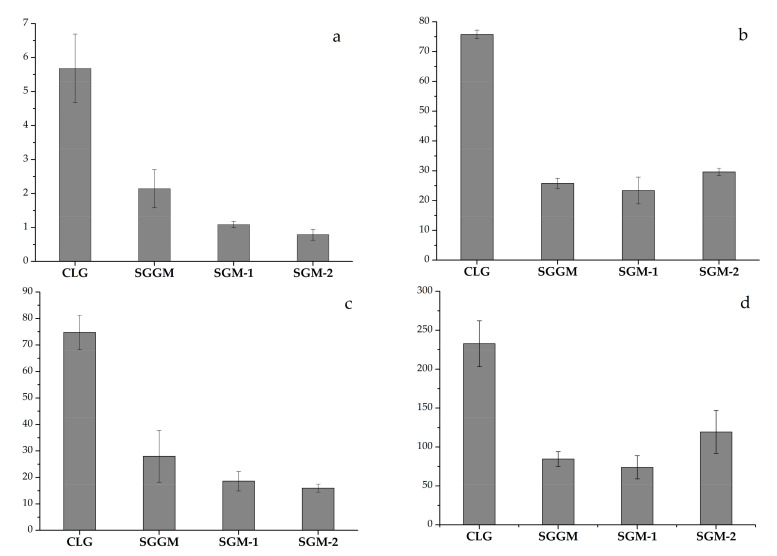
Effect of GGM/GM sulfates (1.67 mg/mL) on the collagen-induced human platelet aggregation (*n* = 4). (**a**) Lag phase in the platelet aggregation curve, min; (**b**) platelet aggregation, %; (**c**) slope of the platelet aggregation curve, rel. units/min; and (**d**) area under the platelet aggregation curve, rel. units × min.

The inhibition of GM and GM sulfates in the collagen-induced platelet aggregation is important since collagen is involved in the very first phases of platelet activation and adhesion (Figure 5).

## 4. Conclusions

Sulfates of natural gel-like galactomannan and galactoglucomannan polysaccharides were synthesized using chlorosulfonic or sulfamic acid in pyridine and in a sulfamic acid-urea deep eutectic solvent.

The effect of the process conditions on the sulfur content in the resulting polysaccharide sulfates was shown.

The introduction of a sulfate group into polysaccharide molecules was proven by the IR spectroscopy investigations. During sulfation, the absorption bands appeared in the FTIR spectra of the reaction products in the region of 1245–1255 cm^−1^, which corresponds to the sulfate group vibrations. The molecular weight distribution for the obtained gel-like natural polysaccharide sulfates was studied by gel-permeation chromatography.

Understanding of the anticoagulant action of galactomannan/galactoglucomannan sulfates and the inhibitory activity against the collagen-induced platelet aggregation in the two investigated samples (SGGM and SGM-2), along with the inhibition of the ADP-induced platelet aggregation, can serve as an additional factor in selecting the compounds promising for the polymer surface modification in medical devices, which ensures their compatibility with blood and thromboresistance.

## Figures and Tables

**Table 1:** Effect of the GM and GGM sulfation conditions on the sulfur content in the reaction products.

Sample No.	Time, h	Temperature, °C	Sulfur Content, wt %	
			GM	GGM
1	2.5	90	13.2	16.5
2	2.5	80	11.9	13.1
3	2.5	70	9.2	7.9
4	1.5	90	12.1	13.6
5	1.5	80	11.1	10.4

**Table 2:** Structural parameters of the GGM and GM sulfates.

Sample	Conditions	Molecular Weight, kDa	Polydispersity Index	Sulfur Content, %	Degree of Sulfation (DS)
SGGM	Sulfamic acid + urea in pyridine	15.6	1.44	16.4	1.77
SGM-1	Chlorosulfonic acid in pyridine	192.5	2.75	13.8	1.25
SGM-2	Deep eutectic solvent (Sulfamic acid + urea)	276.8	4.78	11.2	0.88

## Data Availability

Not applicable.
